# The basal transcription factor II H subunit Tfb5 is required for stress response and pathogenicity in the tangerine pathotype of *Alternaria alternata*

**DOI:** 10.1111/mpp.12982

**Published:** 2020-08-10

**Authors:** Huilan Fu, Kuang‐Ren Chung, Yunpeng Gai, Lijuan Mao, Hongye Li

**Affiliations:** ^1^ Key Laboratory of Molecular Biology of Crop Pathogens and Insects Institute of Biotechnology Zhejiang University Hangzhou China; ^2^ Department of Plant Pathology College of Agriculture and Natural Resources National Chung‐Hsing University Taichung Taiwan; ^3^ Analysis Center of Agrobiology and Environmental Sciences Faculty of Agriculture, Life and Environment Sciences Zhejiang University Hangzhou China

**Keywords:** ACT toxin, *Alternaria alternata*, cutinase, DNA damage, oxidative stress, TFIIH, virulence

## Abstract

The basal transcription factor II H (TFIIH) is a multicomponent complex. In the present study, we characterized a TFIIH subunit Tfb5 by analysing loss‐ and gain‐of‐function mutants to gain a better understanding of the molecular mechanisms underlying stress resistance and pathogenicity in the citrus fungal pathogen *Alternaria alternata*. Tfb5 deficiency mutants (Δ*Aatfb5*) decreased sporulation and pigmentation, and were impaired in the maintenance of colony surface hydrophobicity and cell wall integrity. Δ*Aatfb5* increased sensitivity to ultraviolet light, DNA‐damaging agents, and oxidants. The expression of *Aatfb5* was up‐regulated in the wild type upon infection in citrus leaves, implicating the requirement of *Aatfb5* in fungal pathogenesis. Biochemical and virulence assays revealed that Δ*Aatfb5* was defective in toxin production and cellwall‐degrading enzymes, and failed to induce necrotic lesions on detached citrus leaves. *Aatfb5* fused with green fluorescent protein (GFP) was localized in the cytoplasm and nucleus and physically interacted with another subunit, Tfb2, based on yeast two‐hybrid and co‐immunoprecipitation analyses. Transcriptome and Antibiotics & Secondary Metabolite Analysis Shell (antiSMASH) analyses revealed the positive and negative roles of *Aatfb5* in the production of various secondary metabolites and in the regulation of many metabolic and biosynthetic processes in *A. alternata. Aatfb5* may play a negative role in oxidative phosphorylation and a positive role in peroxisome biosynthesis. Two cutinase‐coding genes (*AaCut2* and *AaCut15*) required for full virulence were down‐regulated in Δ*Aatfb5*. Overall, this study expands our understanding of how *A. alternata* uses the basal transcription factor to deal with stress and achieve successful infection in the plant host.

## Graphical abstract

This study expands our understanding of how the tangerine pathotype of *Alternaria alternata* uses the TFIIH subunit Tfb5 to deal with stress and achieve successful infection in the plant host.

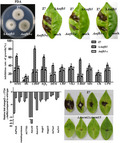

## 1 INTRODUCTION

The basal transcription factor II H (TFIIH) is a protein complex commonly found in different kingdoms, including animals, yeasts, plants, and protists (Compe and Egly, 2016; Rimel and Taatjes, 2018). The TFIIH complex consists of 10 subunits, which are grouped into two subcomplexes: a core TFIIH with an assembly of seven subunits including Ssl2, Rad3, Tfb1, Tfb2, Ssl1, Tfb4, and Tfb5, and a cyclin‐dependent kinase‐activating kinase (CAK) subcomplex comprising Tfb3, Ccl1, and Kin28 (Kainov *et al*., 2010). The TFIIH complex regulates a wide array of cell processes, including nucleotide excision repair (NER), cell cycle regulation, E3 ubiqutin ligase activity, cullin neddylation, and chromosome segregation (Compe and Egly, 2016). In humans, mutations in TFIIH subunits decrease the ability of NER, causing severe developmental defects such as Cockayne syndrome (CS), xerodermapigmentosum (XP), and trichothiodystrophy (TTD) (Lehmann, 2003). The NER machinery stabilizes the genome by removing DNA damage induced by ultraviolet (UV) light or chemicals (Hoeijmakers, 2001). There are two types of NER: global genome NER and transcription‐coupled NER (TC‐NER) (Ding *et al*., 2007). The TC‐NER is activated when RNA polymerase encounters DNA damage, and TFIIH is one of the important components of the RNA polymerase II complex (Ding *et al*., 2007; Rimel and Taatjes, 2018).

The *tfb5* gene, originally identified in yeasts, encodes a small peptide that is highly conserved throughout evolution (Ranish *et al*., 2004). Tfb5 interacts with other subunits to stabilize and ensure the proper function of the entire TFIIH complex (Kainov *et al*., 2010). In yeasts, Tfb5 forms a heterodimer with Tfb2, both of which share similar structures (α/β split with C‐terminal helix) (Theil *et al*., 2014; Compe and Egly, 2016). Interaction between Tfb5 and Tbf2 has been confirmed in yeasts and human cells (Zhou *et al*., 2007; Nonnekens *et al*., 2013). Studies on the function of Tfb5 in *Drosophila* and mouse systems have revealed similar roles in cell development and DNA damage responses (Aguilar‐Fuentes *et al*., 2008; Theil *et al*., 2013). In yeasts, Tfb5 is required for growth and UV irradiation repair and maintenance of the stability of the TFIIH complex (Zhou *et al*., 2007). In the rice blast fungus *Magnaporthe oryzae*, *tfb5* is required for conidiation and redox resistance, and its expression is down‐regulated in a circadian‐*Twilight* (*twl*) mutant (Deng *et al*., 2015). Despite the pioneering work in other species, the function of Tfb5 in *Alternaria alternata* remains unclear.

The necrotrophic fungal pathogen *A. alternata* is capable of infecting many economically important crops and resulting in yield losses. Some strains of *A. alternata* produce mycotoxins causing food contamination. Several pathotypes of *A. alternata* produce the host‐selective toxin (HST) that acts as a pathogenicity factor to kill host cells before colonization (Tsuge *et al*., 2013; Meena *et al*., 2017). Alternaria brown spot of citrus, caused by the tangerine pathotype of *A. alternata*, is one of the important diseases on citrus worldwide. This disease mainly affects tangerines, grapefruit, and their hybrids (Akimitsu *et al*., 2003). The pathogen initiates its infection by secreting an ACT (*A. alternata f. sp. citri tangerine*) toxin, which shares a common 9,10‐epoxy‐8‐hydroxy‐9‐methyl‐decatrienoic acid with that of AK (*A. alternata* f. sp.* kikuchana*) and AF (*A. alternata* f. sp.* fragariae*) toxin produced by the Japanese pear and strawberry pathotypes of *A. alternata*, respectively (Kohmoto, 1993; Imazaki *et al*., 2010). Genes encoding polypeptides involved in the biosynthesis of ACT toxin are clustered and located in a small, conditionally dispensable chromosome (Akamatsu *et al*., 1999; Ajiro *et al*., 2010; Tsuge *et al*., 2013).

In addition to ACT toxin, the ability to produce cell wall‐degrading enzymes (CWDEs) is important for successful penetration and colonization of *A. alternata* in citrus. CWDEs play important roles in the virulence of some plant pathogenic fungi as they rely on CWDEs to degrade plant cell walls (Carvalho *et al*., 1999). Cutinase has recently been demonstrated to be required for full virulence in the tangerine pathotype of *A. alternata* (Ma *et al*., 2019). A fungal strain deleted at both cutinase‐encoding genes (*Aacut3* and *Aacut7*) has decreased cutinase activity and reduced virulence on unwounded citrus leaves. Moreover, *A. alternata* has to cope with toxic reactive oxygen species (ROS) in order to gain successful colonization in citrus leaves. Several proteins, including the NADPH oxidase (Yang and Chung, 2013), the redox‐responsive transcription factor Yap1 (Lin *et al*., 2009), the mitogen‐activated protein (MAP) kinase Hog1 (Lin and Chung, 2010), the stress response regulator Skn7 (Chen *et al*., 2012), and proteins in the thioredoxin and glutaredoxin systems (Ma *et al*., 2018), have been demonstrated to be required for resistance to ROS and full virulence of *A. alternata* on citrus. It has been hypothesized that H_2_O_2_ generated by the NADPH oxidase complex acts as a secondary messenger to activate the expression of *Yap1* and *Hog1*, which activate both thioredoxin and glutaredoxin systems for ROS detoxification (Ma *et al*., 2018).

The filamentous phytopathgenic fungus *A. alternata* has a different lifestyle, gene regulation complexity, and metabolic diversity from yeasts. In this study, we explore the function of a *Tfb5* ortholog in *A. alternata*. Our results demonstrate that *A. alternata tfb5* (*Aatfb5*) is required for resistance to DNA‐damaging and oxidative stress, maintenance of surface hydrophobicity, and cell wall integrity, as well as full virulence.

## 2 RESULTS

### 2.1 Identification and deletion of *Aatfb5*

The sequence of the *tfb5* gene (accession number MT184174) in the tangerine pathotype of *A. alternata* Z7 (*Aatfb5*) was obtained from its genome data (GCA 001572055.1). The *Aatfb5* gene encodes a polypeptide sharing 37% amino acid identity with that of the *Saccharomyces cerevisiae tfb5* gene (*Sctfb*; KZV12316.1). Phylogenetic and domain analyses confirmed the orthology of *Aatfb5* with other *tfb5* genes (Figure S1a). Sequence alignment between *Aatfb5* and *Sctfb5* indicated that both proteins have characteristic stretches of hydrophobic residues (Figure S1b). The *Aatfb5* gene was found to contain a 326‐bp open reading frame (ORF) interrupted by two small introns of 47 and 60 bp, which can be translated into a protein of 72 amino acids.

To explore the function of *Aatfb5*, we generated deletion mutants using the homology recombination strategy (Figure S1c). Candidate mutants were identified by PCR and successful disruption of *Aatfb5* in two mutants (designated Δ*Aatfb5‐1* and Δ*Aatfb5‐4*) was further confirmed by Southern blotting (Figure S1d,e). Because the two disrupted mutants were similar in terms of radial growth, sporulation, and pathogenicity, Δ*Aatfb5‐1* was used in the study. A complementation strain designated *Aatfb5‐c* was generated by transferring a functional copy of *Aatfb5* into protoplasts prepared from Δ*Aatfb5*. The complementation strain was also examined by PCR and the presence of the full‐length *Aatfb5* gene in the *Aatfb5‐c* mutant was confirmed by Southern blotting (Figure S1d,e).

### 2.2 *Aatfb5* is involved in fungal development

**FIGURE 1:**
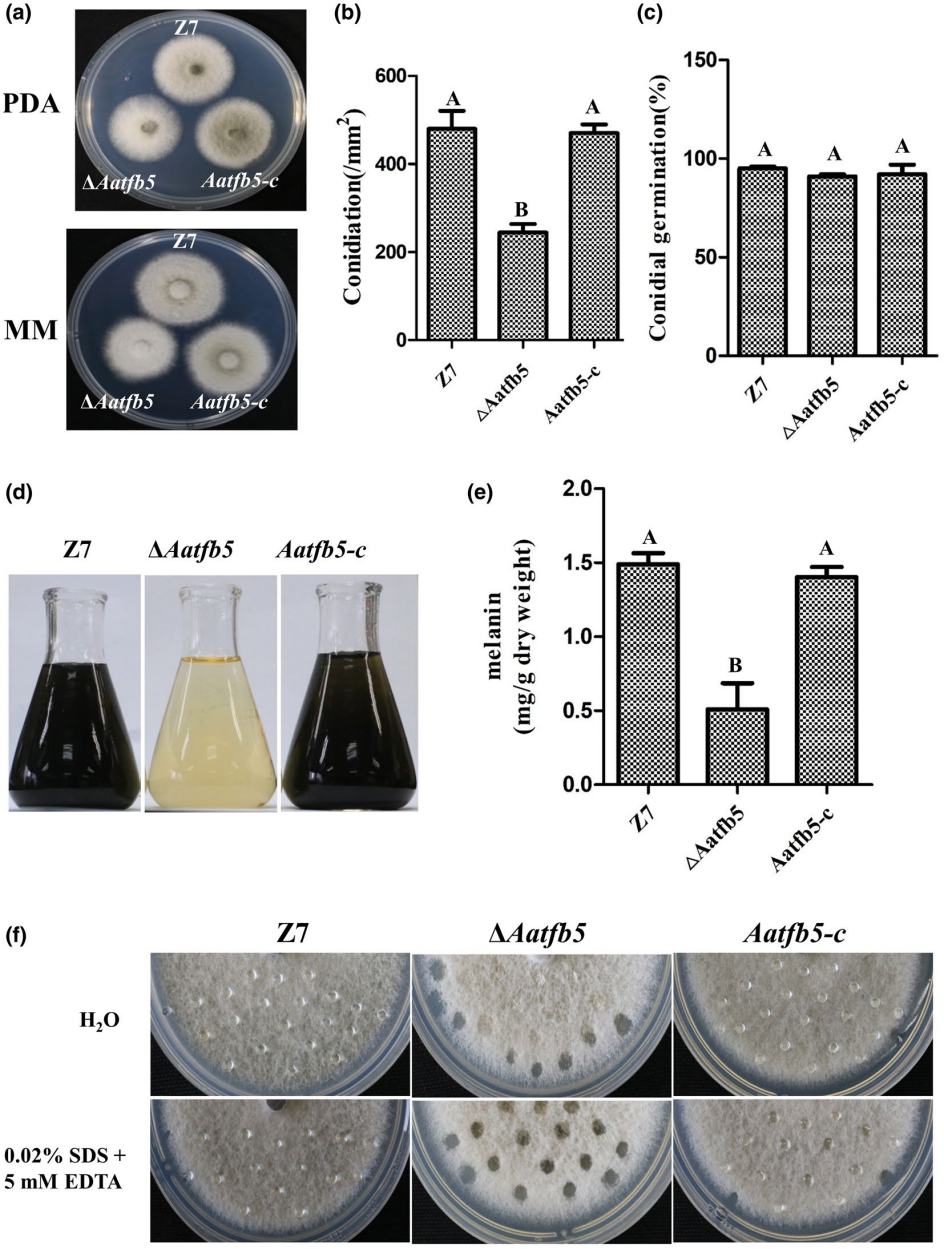
Aatfb5 is required for radial growth, spore formation, pigmentation, and surface hydrophobicity. (a) Colonies of *Alternaria alternata* strains grown on potato dextrose agar (PDA) and minimal medium (MM). (b) Quantitative analysis of conidia produced by *A. alternata* strains. Δ*Aatfb5*, *Aatfb5‐c*, and the wild type (Z7) were grown on V8 juice agar at 26°C for 8 days. (c) Conidial germination rate of *A. alternata* strains. Conidial suspensions were sprayed on water agar and incubated at 26°C for 12 hr. (d) Pigment accumulation in *A. alternata* strains grown in potato dextrose broth. (e) Quantification of melanin in Z7, Δ*Aatfb5*, and *Aatfb5‐c*. Means indicated by the same letter in a panel are not significantly different from one another, *p* < .05. (f) Surface hydrophobicity of Z7, Δ*Aatfb5*, and *Aatfb5‐c* was assessed by placing 10 μl of water or solution containing 0.02% SDS and 0.5 mM EDTA on the colony surface

Deletion of *Aatfb5* reduced fungal radial growth by 23% on minimal medium (MM) but maintained wild‐type growth on potato dextrose agar (PDA; Figure 1a). When grown on V8 juice medium, conidial production was reduced in Δ*Aatfb5* by c.50% compared with the wild type at 8 days of incubation. Sporulation was restored in the *Aatfb5‐c* strain (Figure 1b). Germination rates of spores from the wild type, Δ*Aatfb5*, and *Aatfb5‐c* strains were similar (Figure 1c).

### 2.3 *Aatfb5* is involved in melanin synthesis and surface hydrophobicity

Δ*Aatfb5* reduced pigmentation dramatically when cultured on PDA or in potato dextrose broth (PDB; Figure 1d). Quantification of melanin revealed that Δ*Aatfb5* produced only c.33% melanin compared to the wildtype and the *Aatfb5‐c* strains (Figure 1e). Colony surface hydrophobicity assays showed that water drops could remain on the surface of mycelium of the wild type for more than 12 hr (Figure 1f). A solution containing sodium dodecyl sulphate (SDS) and ethylenediaminetetraacetic acid (EDTA) remained on the surface of the wild‐type colony for about 30 min before soaking into the mycelium. In contrast, Δ*Aatfb5* mycelium failed to retain water as effectively as the wild type, producing wet spots on the colony surface, particularly in the presence of SDS and EDTA. *Aatfb5‐c* displayed a similar ability to retain water drops on the colony surface to the wild type.

### 2.4 *Aatfb5* is required for resistance to DNA‐damaging, oxidizing, and cell wall‐interfering agents

**FIGURE 2:**
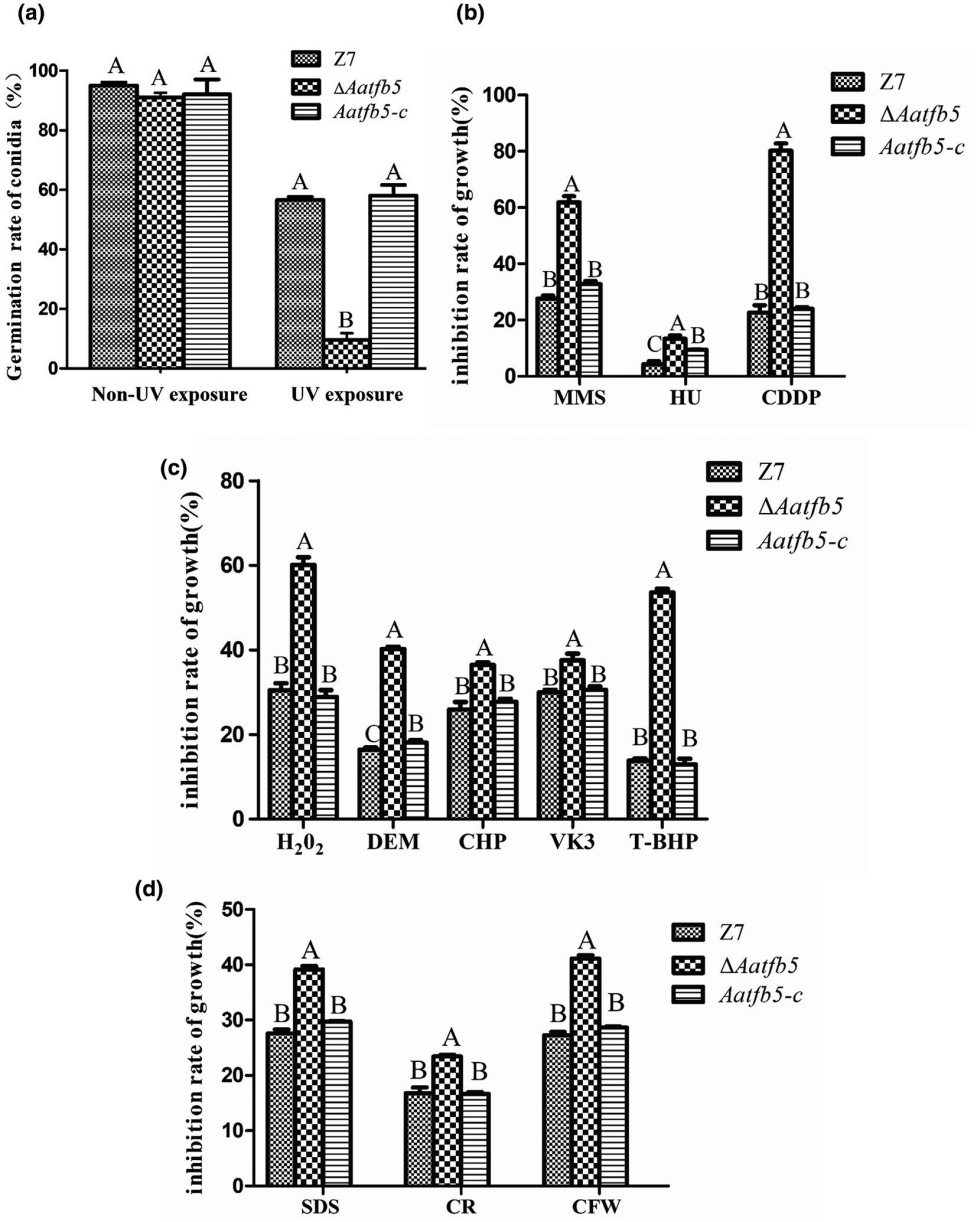
Tfb5 is required for stress resistance. (a) Germination rates of conidia on water agar plates after being exposed to UV light (254 nm, 100 J/m^2^) for 15 s. (b) Growth inhibition rates of the strains cultured on minimal medium (MM) plates containing 0.02% methyl methanesulphonate (MMS), 20 mM hydroxyurea (HU), or 0.1 mM cisplatin (CDDP). (c) Growth inhibition rates of the strains cultured on MM plates containing 10 mM hydrogen peroxide (H_2_O_2_), 0.05% diethyl maleate (DEM), 0.01% cumylhydroperoxide (CHP), 2 mM menadione (vitamin K3, VK3), or 0.05% *tert*‐butyl‐hydroxyperoxide (T‐BHP). (d) Growth inhibition rates of the strains cultured on MM plates containing 100 µg/ml sodium dodecyl sulphate (SDS), 100 µg/ml Congo red (CR), or 200 µg/ml calcofluor white (CFW). Sensitivity tests were conducted by growing fungal strains on MM amended with a chemical. Means indicated by the same letter in a panel are not significantly different from one another, *p* < .05

As mentioned above, spores of Δ*Aatfb5* germinated as effectively as those of the wild type. On exposure to UV light (254 nm, 100 J/m^2^) for 15 s, spores produced by Δ*Aatfb5* germinated at rates slower than those of the wild type, reducing by 10%. *Aatfb5‐c* spores germinated at rates comparable to those of wild type (Figure 2a). Chemical sensitivity assays on MM revealed that Δ*Aatfb5* increased sensitivity to DNA‐damaging agents methanesulphonate (MMS, 0.02%), hydroxyurea (HU, 20 mM), and cisplatin (CDDP, 0.1 mM). *Aatfb5‐c* displayed wild‐type sensitivity to DNA‐damaging agents (Figures 2b and S2a).

Compared with the wild type, Δ*Aatfb5* increased sensitivity to hydrogen peroxide (H_2_O_2,_ 10 mM), diethyl maleate (DEM, 0.05%), cumyl hydroperoxide (CHP, 0.01%), menadione (VK3, 2 mM), and *tert*‐butyl‐hydroxyperoxide (T‐BHP, 0.05%) (Figures 2c and S2b). *Aatfb5‐c* showed wild‐type resistance to those compounds.

As assayed on MM, Δ*Aatfb5* significantly increased sensitivity to sodium dodecyl sulphate (SDS, 100 µg/ml), Congo red (CR, 100 µg/ml), and calcofluor white (CFW, 200 µg/ml) compared to the wild type. The complementation mutant *Aatfb5‐c* displayed wild‐type sensitivity to cell wall‐interfering agents (Figures 2d and S2c), indicating that *Aatfb5* is involved in the maintenance of cell wall integrity.

### 2.5 *Aatfb5* is required for full virulence

**FIGURE 3:**
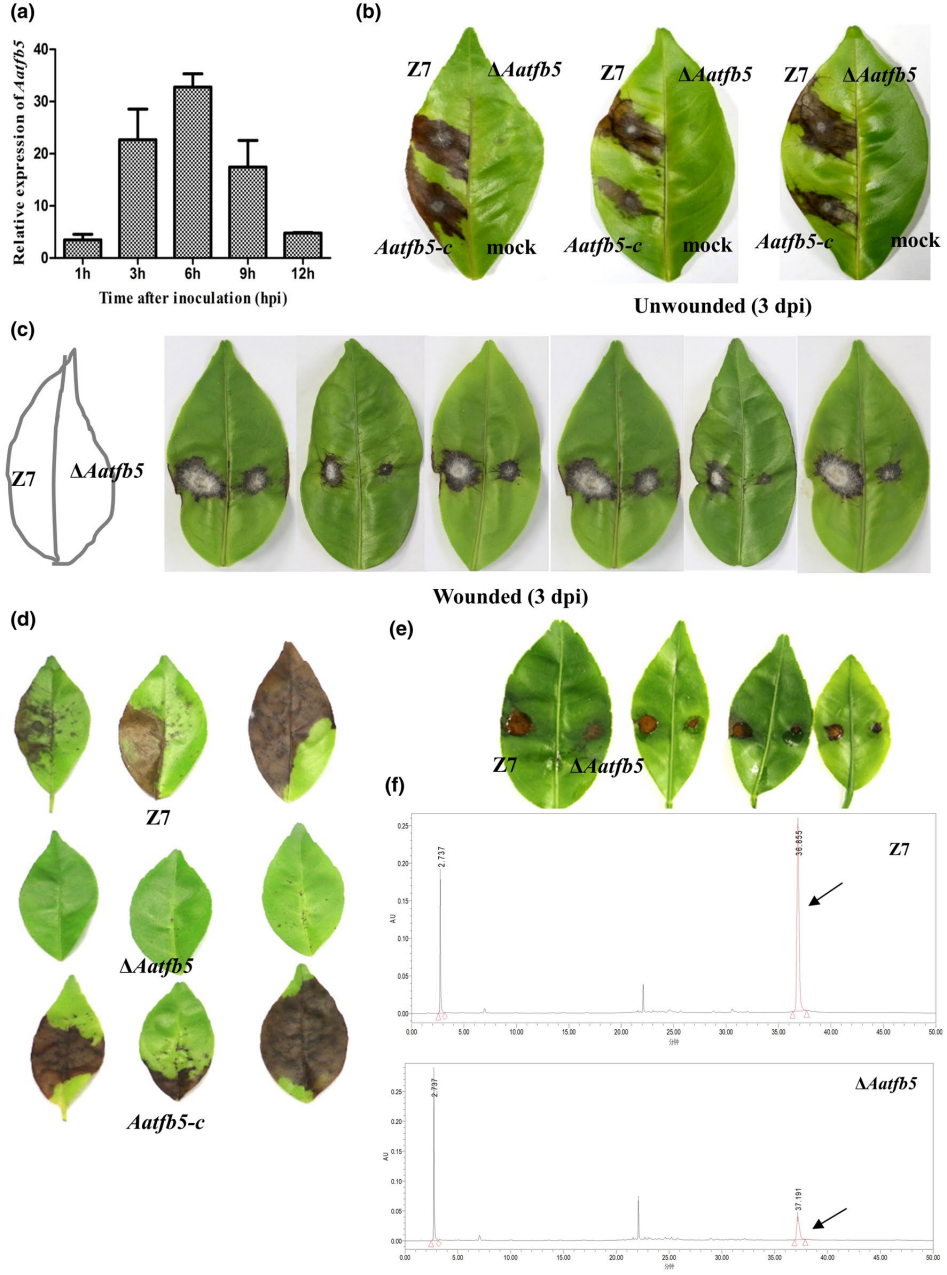
Tfb5 is required for *Alternaria alternata* pathogenesis to citrus leaves. (a) Expression of *Aatfb5* in the wild‐type strain inoculated to detached citrus Hongjv leaves. RNA was purified every 3 hr postinoculation (hpi), and used for cDNA synthesis and quantitative reverse transcription PCR analysis. (b) Fungal pathogenicity was assessed on detached Hongjv leaves (unwounded) inoculated by placing 10 µl of conidial suspensions (10^4^ conidia/ml) prepared from wild type (Z7), Δ*Aatfb5*, and *Aatfb5‐c* on each spot. Spots treated with water were used as mock controls. (c) Fungal pathogenicity was assessed on detached Hongjv leaves that were wounded with a fine needle before inoculation. Leaves were inoculated with 10 µl of conidial suspensions (10^4^ conidia/ml) prepared from Z7, Δ*Aatfb5*, and *Aatfb5‐c* on each spot. (d) Fungal pathogenicity was assessed by uniformly spraying conidial suspensions onto detached Hongjv leaves. Inoculated leaves were kept in a plastic box for 3 days for lesion development. (e) A leaf necrosis assay for the toxicity of ACT toxin by placing cell‐free culture filtrates from the wild type or Δ*Aatfb5* on Hongjv leaves that were wounded with a fine needle before treatment. (f) HPLC analysis of ACT toxin purified from culture filtrates of Z7 and Δ*Aatfb5*. ACT toxin is indicated by an arrow

Quantitative RT‐PCR (RT‐qPCR) analysis revealed that the expression level of *Aatfb5* in the wild type increased by as much as 20‐fold 3 hr postinoculation (hpi) and by 30‐fold 6 hpi, then gradually decreased 9 hpi (Figure 3a), implying the involvement of *Aatfb5* in pathogenesis. Pathogenicity tests using point inoculation by placing 10 μl spore suspensions (10^4^ spores/ml) on the surface of citrus leaves revealed that Δ*Aatfb5* failed to induce necrotic lesions 2 days postinoculation (dpi) on unwounded Hongjv leaves, while both the wild type and *Aatfb5‐c* induced necrotic lesions (Figure 3b). When citrus leaves were wounded before inoculation, Δ*Aatfb5‐*induced necrotic lesions, but the sizes of lesions were much smaller than those induced by the wild type (Figure 3c). Pathogenicity tests using spray inoculation of spore suspension also revealed that Δ*Aatfb5* failed to induce lesions on detached citrus leaves (Figure 3d).

### 2.6 *Aatfb5* is required for ACT toxin biosynthesis

Fungal strains (wild type and Δ*Aatfb5*) were cultured in Richard's medium for 24 days for ACT toxin production. Cell‐free culture filtrates were collected by passing through cheesecloth and a 0.45 µm filter, and used for bioassays on detached Hongjv leaves that were wounded before inoculation. The results reveal that culture filtrates collected from Δ*Aatfb5* resulted in smaller lesions compared to those induced by culture filtrates of the wild type (Figure 3e). HPLC analysis of culture filtrates confirmed further that Δ*Aatfb5* produced a smaller quantity of ACT toxin than the wild type (Figures 3f and S3).

### 2.7 Deletion of *Aatfb5* affects cutinase activity

**FIGURE 4:**
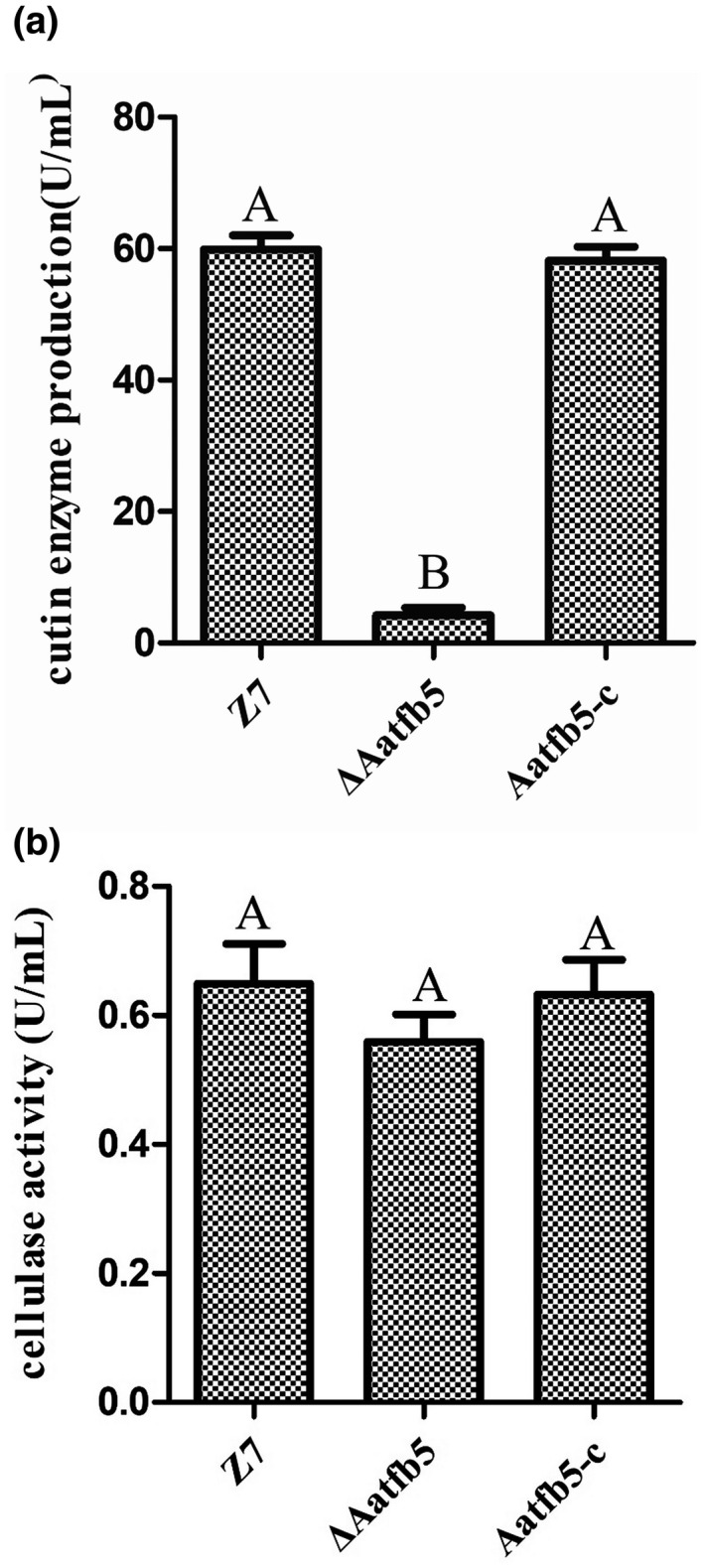
Tfb5 is required for cell wall‐degrading enzymes. (a) Quantification of cutinase activity in wild type (Z7), Δ*Aatfb5*, and *Aatfb5‐c*. (b) Quantitative analysis of cellulase activities in Z7, Δ*Aatfb5*, and *Aatfb5‐c*. Means indicated by the same letter are not significantly different from one another, *p* < .05

To test if Aatfb5 is involved in cell wall‐degrading enzyme activities, fungal strains (Z7, Δ*Aatfb5*, and *Aatfb5‐c*) were cultured in modified Czapek's medium amended with inducers for 24 hr. Culture filtrates were mixed with *p*‐nitrobenzoic acid (for cutinase activities) or dinitrosalicylic acid (for cellulases) containing reagents and measured using a spectrophotometer. The results indicated that Δ*Aatfb5* produced significantly lower amounts of cutinase than the wild type and *Aatfb5‐c* (Figure 4a). Δ*Aatfb5* produced slightly lower amounts of cellulase than the wild type and *Aatfb5‐c* (Figure 4b).

### 2.8 Subcellular localization and interaction pattern of Aatfb5

**FIGURE 5:**
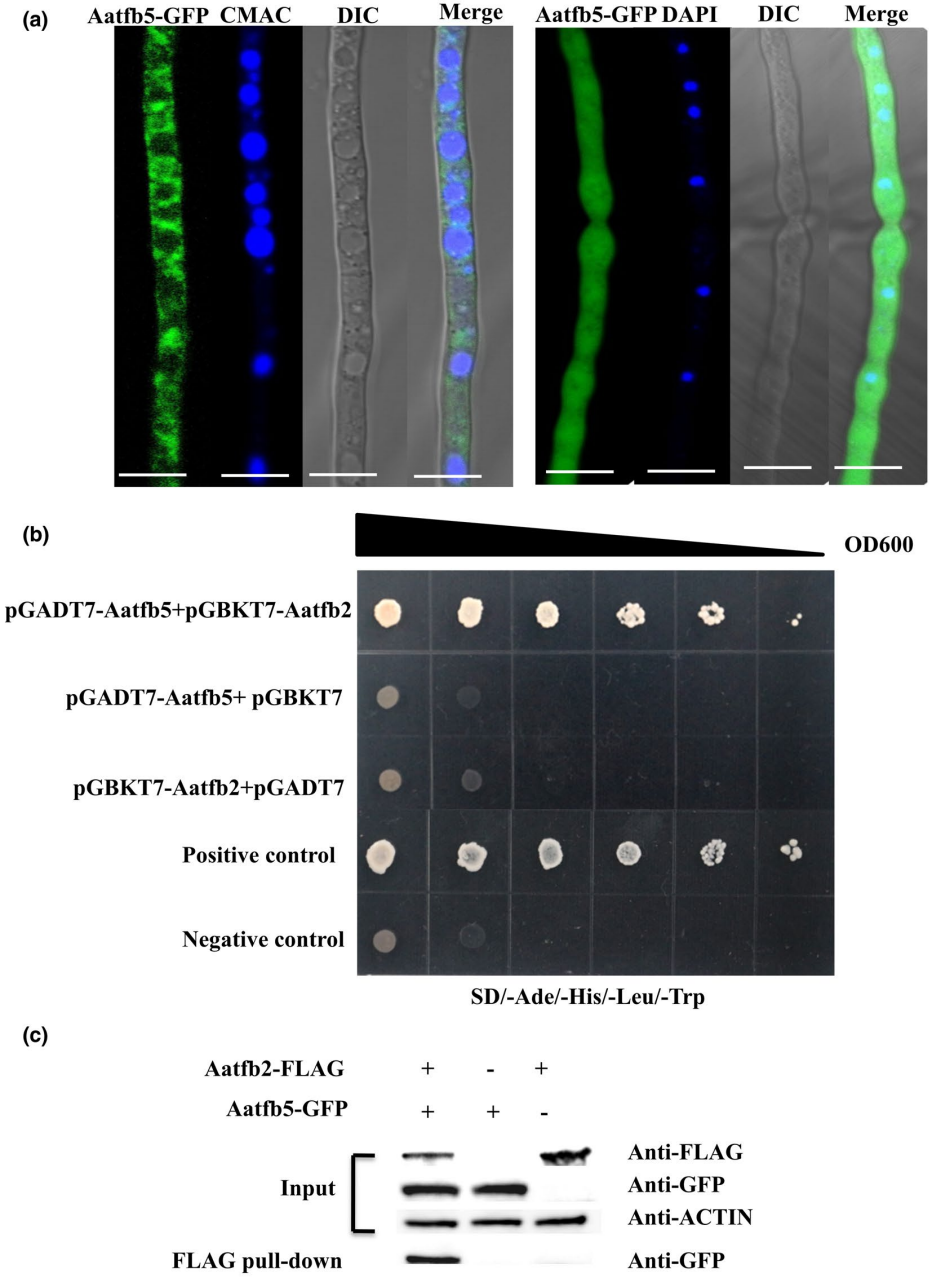
Localization and interaction of Aatfb5 in *Alternaria alternata*. (a) Aatfb5‐GFP is localized in the nucleus and cytoplasm but excluded from vacuoles. Nuclei were stained with 4′,6‐diamidino‐2‐phenylindole (DAPI). Fungal vacuoles were stained with 7‐amino‐4‐chloromethylcoumarin (CMAC). Scale bars, 5 µm. (b) Yeast two‐hybrid analysis reveals the interaction between Aatfb5 and Aatfb2. Serial dilutions of yeast cells (cells/ml) transferred with the bait and prey constructs indicated in the figure were assayed for growth on SD/−Ade/−His/−Leu/−Trp plates. The pGBKT7‐53 and pGADT7 pair was used as a positive control and the pGBKT7‐LAM and pGADT7 pair was used as a negative control. (c) Co‐immunoprecipitation analysis of Aatfb5‐GFP and Aatfb2‐FLAG in *A. alternata*

A functional Aatfb5 was fused with green fluorescent protein (GFP) and expressed under control of its endogenous promoter in Δ*Aatfb5*, revealing a wide distribution of green fluorescence in the cytoplasm and nucleus except vacuoles (Figure 5a). Given that tfb5 has been found to interact with the TFIIH complex subunit tfb2 in *S. cerevisiae* (Zhou *et al*., 2007), we conducted yeast two‐hybrid (Y2H) and co‐immunoprecipitation (Co‐IP) assays to examine whether or not Aatfb5 interacts with Aatfb2 (accession number XM 018525809) in *A. alternata*. Y2H assays revealed that Aatfb5 interacted with Aatfb2 (Figures 5b and S4). The interaction of Aatfb5 with Aatfb2 was further confirmed by Co‐IP assays. Both Aatfb2‐FLAG and Aatfb5‐GFP plasmids were cotransformed into Δ*Aatfb5*, and the resulting strain was used for Co‐IP assay. The results indicated that Aatfb5 proteins, after being incubated with the anti‐FLAG agarose and eluted, could be detected by the monoclonal anti‐GFP antibodies (Figure 5c), indicating that Aatfb5 physically interacted with Aatfb2.

### 2.9 Transcriptome analysis unravels the global regulatory role of Aatfb5

**FIGURE 6:**
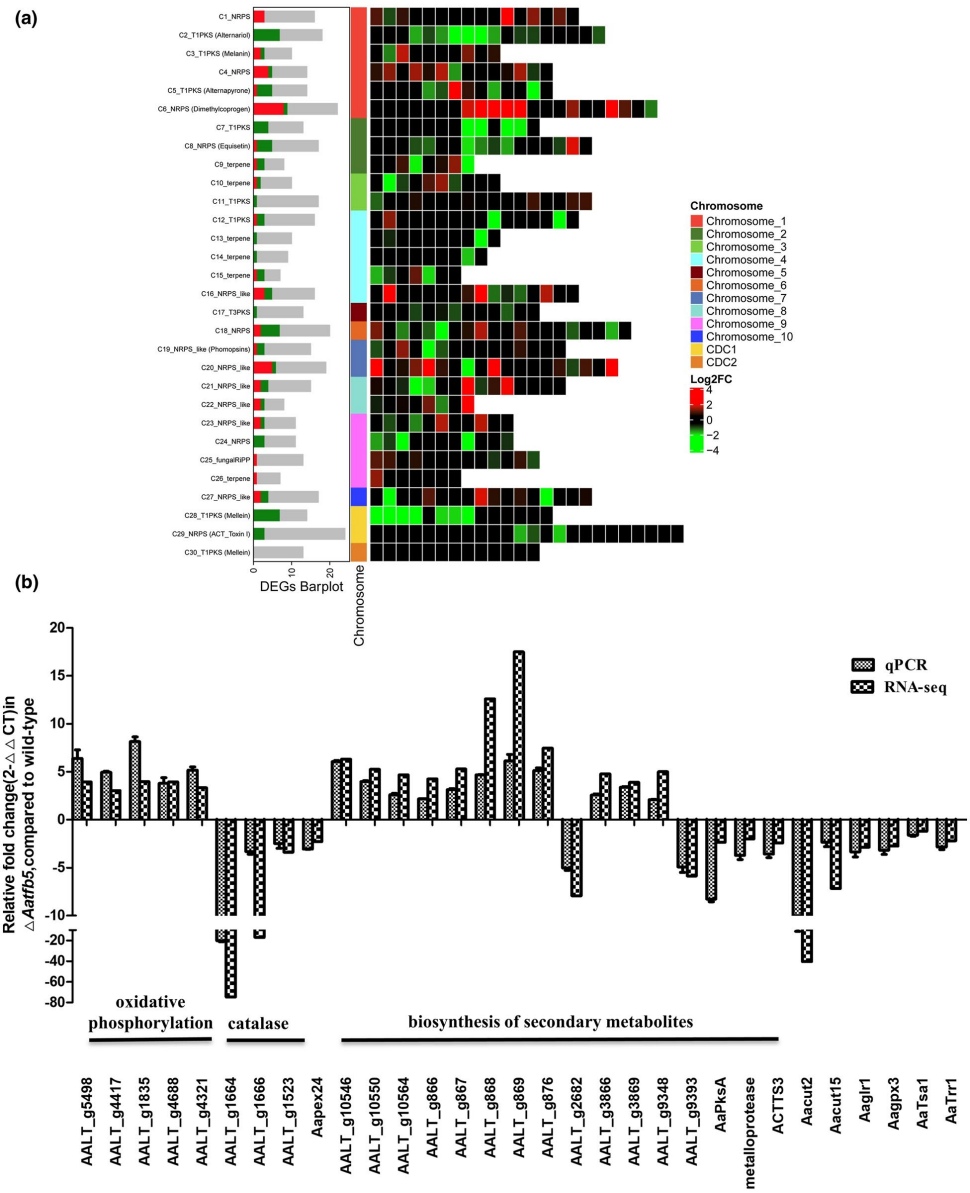
Transcriptome analysis unravels the global regulatory role of Aatfb5. (a) Differential expression of gene clusters associated with the biosynthesis of secondary metabolites in the Δ*Aatfb5* mutant. pks, polyketide synthase; nrps, non‐ribosomal peptide synthetase; t1, type 1; t3, type 3; terpene, terpene synthetase. (b) Relative fold change of 31 genes involved in the oxidative phosphorylation, catalase, peroxin 24, biosynthesis of secondary metabolites, cutinase, and thioredoxin and glutaredoxin systems in Δ*Aatfb5*. The relative transcript levels of target genes were analysed by RNA‐Seq (*p* < .05, log_2_FoldChange > 1). The β*‐*actin coding gene was used as the reference gene. The relative expression level of a gene in Δ*Aatfb5* was determined using a comparative *C*_t_ method in relation to that of the wild type

Transcriptomic analysis was performed to compare the whole‐genome expression profiles between Δ*Aatfb5* and the wild type. Illumina libraries were constructed from Δ*Aatfb5* and the wild type and sequenced using the HiSeq platform. In total, 5,473 differentially expressed genes (DEGs) consisting of 2,673 up‐regulated and 2,800 down‐regulated genes were identified in Δ*Aatfb5* compared to the wild type (Figure S5a). Expression of the genes associated with a conditionally dispensable chromosome was decreased in Δ*Aatfb5* (Figure S5b). Gene ontology (GO) and KEGG pathway enrichment analyses revealed that *Aatfb5* is probably involved in the regulation of many metabolic and biosynthetic processes, and may play a role in oxidative phosphorylation, the tricarboxylic acid (TCA) cycle, and the pentose phosphate pathway (Figure S5c,d). In addition, *Aatfb5* may also regulate the thioredoxin and glutaredoxin system because expression of the genes *AaTrr1* (AAL_g7769), *AaTsa1* (AALT_g1812), *AaGlr1* (AALT_g2809), and *AaGpx3* (AALT_g41) was down‐regulated in Δ*Aatfb5* (Figure 6b).

Biosynthesis gene clusters predicted by antiSMASH 4.0 revealed that Aatfb5 regulates a broad range of gene clusters involved in the biosynthesis of secondary metabolites (Figure 6a and Table S2). Significantly, several genes in cluster 6 were up‐regulated and many genes in clusters 2 (alternariol) and 28 were down‐regulated. Some genes in cluster 3 (melanin) and cluster 29 (ACT toxin) were also down‐regulated in Δ*Aatfb5*.

The expression profiles of some genes observed in transcriptome analysis were validated by RT‐qPCR. The expression levels of five genes associated with oxidative phosphorylation (AALT_g5498, AALT_g4417, AALT_g1835, AALT_g4688, and AALT_g4321) were up‐regulated and four genes associated with peroxisome including three catalases (AALT_g1664, AALT_g1666, and AALT_g1523) and a peroxin 24 (*Aapex24*) were down‐regulated in Δ*Aatfb5* consistent with RNA‐Seq (Figure 6b). RT‐qPCR analysis also confirmed the expression profiles of genes associated with secondary metabolites. RT‐qPCR analysis revealed the down‐regulation of the *AapksA* coding genes involved in the biosynthesis of melanin in ∆*Aatfb5* (Figure 6b). Two genes (*ACTTS3* and a metalloprotease‐coding gene) located in the biosynthesis of ACT toxin cluster were down‐regulated in Δ*Aatfb5* (Figure 6b).

**FIGURE 7:**
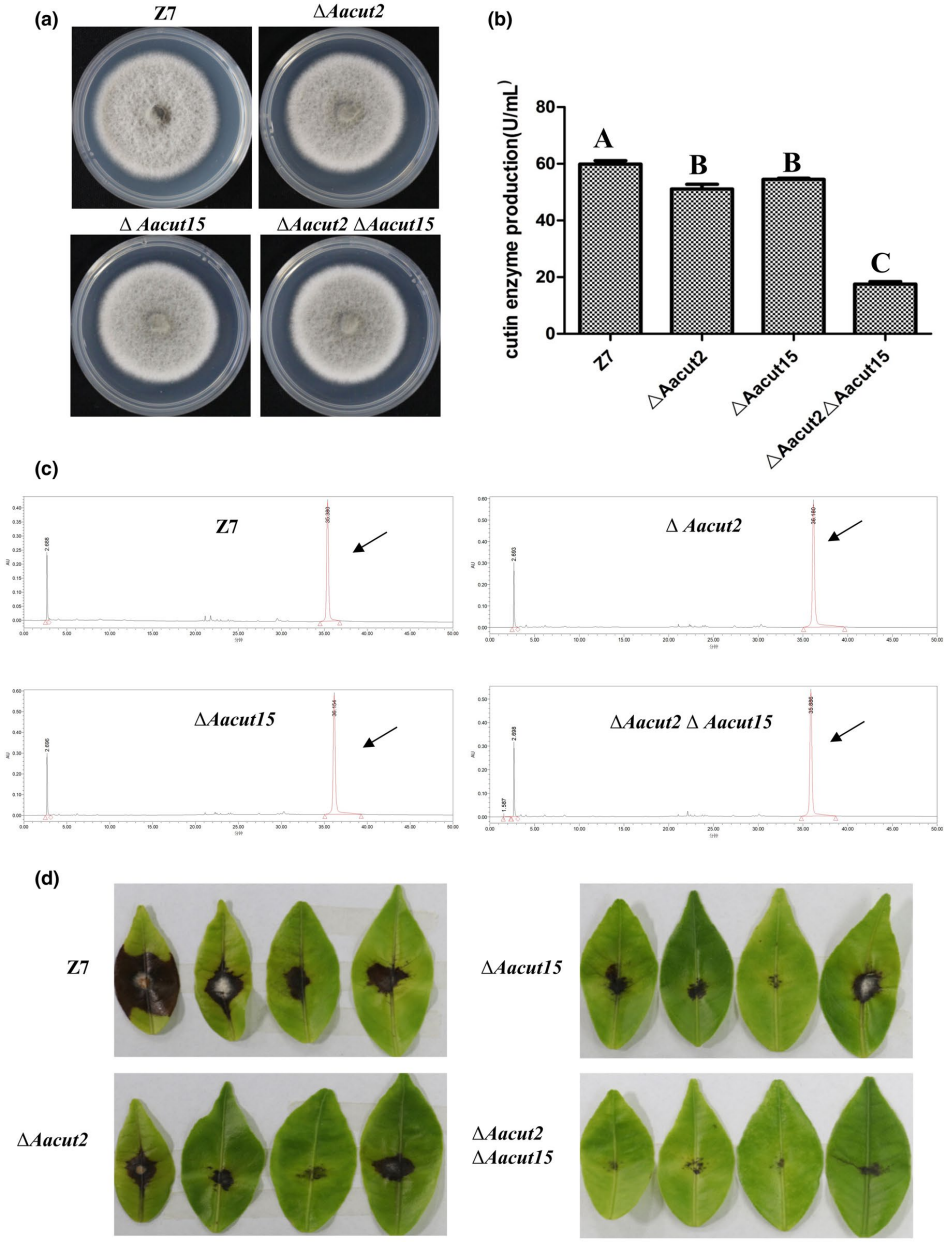
Characteristics of *Aacut2* and *Aacut15* deficiency mutants. (a) Colonies of *Alternaria alternata* strains grown on potato dextrose agar. (b) Quantification of cutinase activity in wild type (Z7), the Cut2 mutant strain (Δ*Aacut2*), the Cut15 mutant strain (Δ*Aacut15*), and the Cut2Cut15 double mutant strain (Δ*Aacut2*Δ*Aacut15*). Means indicated by the same letter are not significantly different from one another, *p* < .05. (c) HPLC analysis of ACT toxin purified from culture filtrates of Z7, Δ*Aacut2*, Δ*Aacut15*, and Δ*Aacut2*Δ*Aacut15*. ACT toxin is indicated by an arrow. (d) Virulence assays on citrus. Hongjv leaves were inoculated by placing 10 µl of conidial suspensions (10^4^ conidia/ml) prepared from Z7 and the Δ*Aacut2*, Δ*Aacut15*, and Δ*Aacut2*Δ*Aacut15*

Transcriptome analysis revealed that the expression of two cutinase‐coding genes, *Aacut2* (AALT_g5432) and *Aacut15* (AALT_g7939), was significantly decreased in Δ*Aatfb5* (Figure 6b). To determine if *Aacut2* and *Aacut15* are involved in virulence in *A. alternata*, the genes were independently or simultaneously deleted in the genome of Z7 (Figure S6a,b). Fungal strains defective in *Aacut2* or *Aacut15* (single gene mutation) or both *Aacut2* and *Aacut15* (double mutation) displayed wild‐type growth, conidiation, and ACT toxin production (Figures 7a,c, S3, and S6c). Assays for cutinase activity indicated that the double mutation strain produced much lower cutinase activity than the wild‐type strain, while deletion of *Aacut2* or *Aacut15* alone had little or no effect on cutinase (Figure 7b). Pathogenicity tests revealed that single gene mutation strains Δ*Aacut2* and Δ*Aacut15*induced necrotic lesions at sizes slightly but significantly smaller than those induced by Z7 as assayed on unwounded Hongjv leaves. Necrotic lesions induced by the double mutation strain Δ*Aacut2*Δ*Aacut15* were significantly smaller than those induced by Z7 at 2 dpi (Figure 7d).

## 3 DISCUSSION

The NER machinery recognizes and removes damage in the DNA double helix in all cells (Hoeijmakers, 2001). Tfb5 is a highly conserved protein involved in cell processes, transcription, and DNA repair in eukaryotes. While Tfb5 is not essential in *A. alternata*, *tfb5* deficiency leads to embryonic lethality in mouse models (Theil *et al*., 2013). In this study, we characterized a yeast *tfb5* homologue in the tangerine pathotype of *A. alternata*. Gain‐ and loss‐of‐function studies revealed that *Aatfb5* is required for growth, conidiation, pathogenicity, maintenance of hyphal surface hydrophobicity, biosynthesis of secondary metabolites, cutinase production, and stress resistance in *A. alternata*. Δ*Aatfb5* reduces growth on MM and shows wild‐type growth in PDB, suggesting that *Aatfb5* is also involved in micronutrient metabolism.

Deletion of *Aatfb5* apparently affects the formation of conidia in *A. alternata*. This observation is consistent with a previous report in the rice blast fungus *M. oryzae* (Deng *et al*., 2015). In *M. oryzae*, a circadian‐regulated Twilight (TWL) gene is required for proper conidiation and pathogenesis. The expression of *Motfb5* is significantly down‐regulated in the Δ*twl* mutant, while overexpression of the *Motfb5* gene restores a sporulation defect in the Δ*twl* mutant and increases sporulation in the wild‐type strain. Furthermore, Tfb5‐GFP fails to translocate to the nucleus in the Δ*twl*, indicating that *Motfb5* is directly regulated by MoTwl (Deng *et al*., 2015). Conidia play an important role in the disease cycle of *A. alternata*. The formation of conidia is a complex process. In *A. alternata*, studies have identified several proteins that are involved in conidiation. These include the mitogen‐activated protein kinases FUS3 (Lin *et al*., 2010) and SLT2 (Yago *et al*., 2011), the G‐protein (Wang *et al*., 2010), the cAMP‐dependent protein kinase (Tsai *et al*., 2013), the calcium‐mediated signalling pathways (Tsai and Chung, 2014), the NADPH oxidase complex (Yang and Chung, 2013), and the COP9 signalosome (Wang *et al*., 2018). However, transcriptome analysis revealed that none of these genes showed significant changes at the transcriptional level in Δ*Aatfb5* (Table S3). Moreover, expression of several genes (G protein‐mediated signalling pathways genes, FluG‐mediated genes, BrlA, AbaA and WetA central regulatory genes, and stuA‐coding genes) required for conidiation in other filamentous fungi (Park and Yu, 2012; Wang *et al*., 2018) were not significantly different between Δ*Aatfb5* and the wild type (Table S3). The results indicate that Aatfb5 may regulate conidiation via a novel yet unidentified pathway in *A. alternata*. Alternatively, Aatfb5 may not regulate conidiation via transcriptional activation.

TFIIH is a pivotal part in NER, a DNA‐repair mechanism that removes DNA lesions caused by various environmental factors (Aguilar‐Fuentes *et al*., 2008). *S. cerevisiae tfb5* mutant cells are moderately sensitive to UV light (Zhou *et al*., 2007). Mouse embryonic cells lacking Tfb5 are highly sensitive to UV light (Theil *et al*., 2013). Sensitivity assays revealed that impairment of *Aatfb5* increased sensitivity to UV light in *A. alternata*, consistent with findings in yeasts and mouse cells (Theil *et al*., 2013, 2014). In addition, the *Aatfb5* deficiency strains showed an increased sensitivity to methyl methanesulphonate, hydroxyurea, and cisplatin. Re‐expression of a copy of functional Aatfb5 in Δ*Aatfb5* restored its sensitivity to wild‐type level, indicating that *Aatfb5* plays a role in protection from DNA damage in the plant pathogenic fungus *A. alternata*.

Melanin has many functions in fungi (Toledo *et al*., 2017). In *M. oryzae* and *Colletotrichum* spp., melanin is essential for the formation of turgor pressure in appressoria, which are absolutely required for penetration of plant cuticles (Babitskaya *et al*., 2000). In *A alternata*, melanin has been shown to be required for protection against UV light, but it is not required for pathogenicity (Kawamura *et al*., 1999; Ramona Fetzner *et al*., 2014). Several genes, including *pksA*, *brm2*, and *CmrA*, have been identified to be required for the biosynthesis of DHN melanin in *A. alternata* (Kimura and Tsuge, 1993; Tseng *et al*., 2011; Ramona Fetzner *et al*., 2014). The *brm2* deletion mutant of the Japanese pear pathotype of *A. alternata* is unable to produce melanin, is hypersensitive to UV light, but displays wild‐type virulence (Kawamura *et al*., 1999). In the current study, we found that deletion of *Aatfb5* resulted in a significant decrease of melanin in Δ*Aatfb5*, and conidia produced by Δ*Aatfb5* were hypersensitive to UV light. RNA‐Seq and RT‐qPCR analyses also revealed that the expression of *pksA* (AALT_g6058), a polyketide synthase‐coding gene that is required for pigment synthesis, was down‐regulated in Δ*Aatfb5*. The results confirm further the involvement of *Aatfb5* in the production of melanin.

In addition to resistance to DNA‐damaging agents, *Aatfb5* was required for resistance to several ROS‐generating compounds such as hydrogen peroxide (H_2_O_2_), diethyl maleate (DEM), cumyl hydroperoxide (CHP), menadione (VK3), and *tert*‐butyl‐hydroxyperoxide (T‐BHP) in *A. alternata*. Similar results have been reported in the tfb5 orthologues in mouse (Theil *et al*., 2013) and *M. oryzae* (Deng *et al*., 2015). The mechanisms underlying ROS resistance have been studied in *A. alternata*. Previous research has suggested that the NADPH oxidase complex generates low‐level H_2_O_2_ that acts as a secondary signal to promote the nuclear translocation of Yap1 and Hog1, and to activate the expression of the genes encoding Yap1, Skn7, and Hog1, which in turn activate the thioredoxin (*AaTsa1* and *AaTrr1*) and glutaredoxin (*AaGpx3* and *AaGlr1*) systems in response to oxidative damage (Ma *et al*., 2018). In the present study, RT‐qPCR revealed that the expression of *AaTrr1*,* AaGpx3*, and *AaGlr1* was significantly down‐regulated in Δ*Aatfb5*. These results indicate that the involvement of *Aatfb5* in resistance to toxic oxidants is probably mediated via the regulation of the thioredoxin and glutaredoxin systems in *A. alternata*.

Pathogenicity assays performed on Hongjv leaves revealed that Δ*Aatfb5* failed to induce necrotic lesions on unwounded citrus leaves, indicating that *Aatfb5* plays a profound role in *A. alternata* pathogenesis. This impairment may be largely due to the fact that Δ*Aatfb5* accumulates less ACT toxin than the wild type. The present study indicates that *Aatfb5* regulates the biosynthesis of secondary metabolites. The ability to produce ACT toxin has been demonstrated to be crucial for pathogenicity in the tangerine pathotype of *A. alternata* (Masunaka *et al*., 2005). The involvement of Aatfb5 in the biosynthesis of ACT is confirmed further based on the reduced expression of the genes (metalloptrotease‐encoding gene and ACTTS3) involved in the biosynthesis of ACT toxin in Δ*Aatfb5*.

Because Δ*Aatfb5* incites necrotic lesions significantly smaller than the wild type on detached leaves that are wounded before inoculation, it appears that Δ*Aatfb5* is impaired for penetration. This could be due to a decreased activity of cell wall‐degrading enzymes. Enzymatic assays revealed that Aatfb5 is required for cutinase production. Cutinase is a hydrolytic enzyme that has been shown to play an important role in virulence in many plant pathogenic fungi (Carvalho *et al*., 1999), such as *Curvularia lunata* (Liu *et al*., 2016), *Fusarium solani* (Rogers *et al*., 1994), and *Pyrenopeziza brassicae* (K. A. Davies, 2000). Disruption of *ClCUT7* in *C. lunata* leads to a significant decrease in virulence only on unwounded maize leaves (Liu *et al*., 2016). In the present study, RNA‐Seq and RT‐qPCR revealed that the expression of two cutinase‐coding genes, *Aacut2* (AALT_g5432) and *Aacut15* (AALT_g7939), was down‐regulated in Δ*Aatfb5*. Single deletion of *Aacut2* or *Aacut15* in Z7 led to a moderate reduction in cutinase activity and virulence, but double deletion of *Aacut2* and *Aacut15* significantly reduced the cutinase activity and virulence on citrus. These results demonstrate that *Aatfb5* is required for the expression of the cutinase‐coding genes and virulence in *A. alternata*. These data verify our recent conclusion that cutinase is required for full virulence in the tangerine pathotype of *A. alternata* (Ma *et al*., 2019).

In the budding yeast, tfb5 interacts with tfb2 to stabilize the architecture of TFIIH and the interaction is important for the function of NER (Zhou *et al*., 2007). In living cells, the split‐GFP system has showed that tfb5 interacts with tfb2, both of which are then incorporated into TFIIH (Nonnekens *et al*., 2013). In the current study, we have verified the interaction of Aatfb5 with Aatfb2 by Y2H and Co‐IP assays in *A. alternata*, indicating that a physical interaction between tfb5 and tfb2 plays an important role in the function of TFIIH. After multiple attempts, we were unable to obtain Aatfb2‐deficient mutants, suggesting that Aatfb2 is essential for cell viability in *A. alternata*.

Peroxisomes carry out different functions in fungi, including metabolic activities, antioxidant defences, and pathogenicity (Pieuchot and Jedd, 2012). Proxins encoded by the *Pex* genes are required for the biogenesis of peroxisomes. The expression of *Aapex24* (AALT_g8342), a yeast Pex24 homologue, is down‐regulated in Δ*Aatfb5*. Pex24 is a peroxisome membrane protein, which is essential for peroxisome assembly in yeasts (Tam *et al*., 2002). Peroxisomes contain abundant catalases, which are involved in scavenging ROS (Taheri and Kakooee, 2017). In the present study, the transcription levels of the genes encoding catalases (AALT_g1664, AALT_g1666, and AALT_g1523) were significantly down‐regulated in Δ*Aatfb5*. Whether or not *Aatfb5* is required for the formation of peroxisomes and catalase activity in *A. alternata* warrants further investigation.

In conclusion, we have demonstrated that the basal transcription factor II H subunit‐coding gene *tfb5* is involved in conidiation, pathogenicity, and resistance to DNA‐damaging and oxidative stress in *A. alternata*. In addition, *Aatfb5* affects the biosynthesis of secondary metabolites and cutinase by regulating the expression of the biosynthetic genes. Our research has established, for the first time, the biological functions of *tfb5* in plant pathogenic fungi.

## 4 EXPERIMENTAL PROCEDURES

### 4.1 Fungal strain and culture conditions

The wild‐type strain Z7 (CGMCC3.18907) of *A. alternata*, originally isolated from a diseased citrus leaf in Zhejiang, China (Huang *et al*., 2015; Wang *et al*., 2016), was used as a parent strain for mutagenesis. Unless otherwise indicated, the fungus was grown on PDA or PDB on a shaker set at 160 rpm at 26°C. Conidia were collected from fungal cultures incubated under fluorescent light for 7 days. All fungal strains used in this study were preserved in 15% glycerol solutions stored at −80°C.

### 4.2 Targeted gene disruption and complementation

The deletion mutants were created using previously described protocols (Wang *et al*., 2018). Briefly, a double joint PCR was used to generate three hybrid DNA fragments (Figure S1). An upstream fragment (540 bp) and a downstream fragment (484 bp) of the *Aatfb5* gene were independently amplified from the Z7 genome and fused with a bacterial phosphotransferase B gene (*HPH*). The fused fragments were transformed into protoplasts prepared from Z7 with polyethylene glycol and CaCl_2_ (Lin and Chung, 2010). Putative transformants were picked from PDA containing 100 μg/ml hygromycin, examined by PCR with specific primers, and confirmed further by Southern blotting assay. For genetic complementation, a full‐length *Aatfb5* gene including its promoter sequences (1,085 bp) and 3′‐UTR sequences (323 bp) was amplified and cloned into the p1300‐NEO plasmid (Wang *et al*., 2015). The resultant plasmid was then introduced into the protoplasts prepared from a Δ*Aatfb5* mutant. Transformants were picked from PDA containing 100 μg/ml G418, verified by PCR, and confirmed further by Southern blotting. The upstream (957 bp) and downstream (519 bp) fragments of the *Aacut2* gene were amplified and fused with a neomycin resistance gene (*NEO*). The upstream (723 bp) and downstream fragments (616 bp) of the *Aacut15* gene were amplified and fused with *HPH*. The fused fragments were independently transformed into protoplasts prepared from Z7 and putative transformants were picked from PDA containing 100 μg/ml G418 or hygromycin. For double deletion mutants, the *Aacut2* fused fragments were transformed into protoplasts prepared from Δ*Aacut15*. Putative transformants were picked from PDA containing 100 μg/ml hygromycin and G418, and examined by PCR with specific primers. All primers used in this study are listed in Table S1.

### 4.3 Phenotypic analysis

Sporulation was assessed by culturing fungal strains on V8 juice agar for 7 days. The spore germination assay was performed by spreading 100 μl spore suspensions (10^5^ spores/ml) on water agar (WA) plate, incubated at 26°C for 12 hr, and examined under a microscope (*n* = 300). Stress tolerance was assayed by transferring mycelium plugs onto minimal medium (MM) (Chung, 2003) amended with DNA‐damaging agents, oxidants, cell wall‐interfering agents, or other indicated chemicals at 26°C for 4 days. The growth inhibition rate was calculated by dividing the colony diameter of each strain grown on medium amended with or without a chemical treatment.

### 4.4 Melanin extraction and measurement

Fungal mycelia cultured in PDB for 7 days were collected after filtration through a filter paper. Pigments were extracted from the filtrated mycelia (0.5 g) after being treated with 10 ml 1 M NaOH and boiled at 100°C for 5 hr. The solution containing boiled mycelia was centrifuged at 5,000 × g for 5 min. The pigment extracts were acidified with 5 M HCl to pH 2.0 and centrifuged at room temperature (6,000 × g, 15 min). The pellet was collected, washed three times with ddH_2_O_2_, and redissolved in 1 M NaOH. The solution was determined spectrophotometrically for absorbance at 459 nm and 1 M NaOH was used as a control (Babitskaya *et al*., 2000).

### 4.5 Cutinase and cellulase assays

Freshly harvested mycelia were added to modified Czapek's medium (1 g glucose, 0.6 g NaNO_3_, 0.6 g K_2_HPO_4_, 0.2 g MgSO_4_, 0.2 g KCl, and 0.1 g FeSO_4_.7H_2_O per litre) amended with 0.1% tomato skins (preboiled in 4 g/L oxalate, 16 g/L ammonium oxalate, pH 3.8) (for cutinase activity) or 1.76% sodium carboxymethyl cellulose (for cellulases) and incubated at 26°C on a shaker for 24 hr (Rocha *et al*., 2008). Culture filtrates were centrifuged at 5,000 × g for 30 min. The supernatants were collected and tested for cutinase activity using *p*‐nitrobenzoic acid (PNB)‐mediated hydrolysis and measured for absorbance (A) at 405 nm with a spectrophotometer as described (Rocha *et al*., 2008). One unit of cutinase was defined by producing 1 μg *p*‐nitrophenol per minute. Cellulase activity was determined using the dinitrosalicylic acid (DNS) method to determine the reduced glucose released from 1% citrus pectin and measured for A_540_ nm with a spectrophotometer as described (Kapat *et al*., 1998; Ma *et al*., 2019). One unit of cellulase activity was defined to liberate 1 μmol glucose from 1% citrus pectin per minute.

### 4.6 ACT toxin assays

ACT toxin was extracted from fungal culture filtrates as described (Kohmoto, 1993). Fungal strains were grown in a 300‐ml Richards’ solution at 26°C for 24 days. Culture filtrates after passing through four layers of cheesecloth were mixed with 10 ml Amberlite XAD‐2 resins and incubated for 2 hr. Amberlite XAD‐2 resins were collected by passing through a filter paper and ACT toxin eluted with 40 ml methanol. ACT toxin was analysed by HPLC as previously described (Ma *et al*., 2019). ACT toxin was separated in an XbridgeTMC18.5 column (4.6 × 250 mm) attached to a Waters 880‐PU HPLC system using methanol/0.1% acetic acid as a mobile phase at a flow rate of 1 ml/min. ACT toxin was detected by a UV detector with absorbance measured at 290 nm. A peak of retention time at 36.9–37.9 min was collected and tested for toxicity on citrus leaves. The toxicity was assessed on detached citrus leaves (*Citrus reticulata* 'Hongjv') by placing 10 μl of serially diluted filtrates on the wounded leaf surface. The treated leaves were incubated in a plastic box at 26°C for 3 days. Development of visible lesions 3 days after treatment was indicative of the presence of ACT toxin.

### 4.7 Virulence assays

Virulence assay was conducted on detached Hongjv leaves as described (Yago *et al*., 2011). Spore suspensions (10^4^ spores/ml) were sprayed or point‐inoculated on detached Hongjv leaves, which were or were not wounded by pricking prior to inoculation. The inoculated leaves were placed in a plastic box at 26°C for 3 days for lesion development. Each strain was tested on 12 leaves and the experiments were conducted at least twice.

### 4.8 Transcriptome analysis

Mycelia of tested strains were cultured in PDB for 2 days, harvested, and ground in liquid nitrogen for RNA isolation. Total RNA was extracted using the Axygen RNA purification kit (Capital Scientific). RNA‐Seq was conducted using three biological replicates of each sample. Sequencing libraries were generated using Ultra RNA Library Prep kit (NEB) and were sequenced using an Illumina HiSeq2000 sequencer platform (Illumina Inc.) to generate 150 bp paired‐end reads. Clean reads were obtained by removing reads containing adapters and low‐quality reads from raw data using Trimmomatic v. 0.36 (Bolger *et al*., 2014). The resulting sequences were aligned to the *A. alternata* Z7 genome using Hisat2 v. 2.0.5 (Kim *et al*., 2015) and the number of reads mapped to each gene was counted by FeatureCounts v. 1.5.0‐p3 (Liao *et al*., 2014). Differential expression between wild type and Δ*Aatfb5* was analysed using the DESeq2 R package (Anders and Huber, 2012). Genes with an adjusted *p* value <.05 and an absolute value of log_2_ fold change (log_2_FC) greater than 1 by DESeq2 were assigned as differentially expressed. ClusterProfiler R package was used to test the statistical enrichment of differential expression genes in KEGG pathways and Gene Ontology (GO) (Yu *et al*., 2012). Gene clusters associated with secondary metabolites were predicted using antiSMASH 4.0 (Blin *et al*., 2017). The DEGs and SM gene clusters were plotted using Circos (Krzywinski *et al*., 2009).

### 4.9 RT‐qPCR analysis

Total RNA was extracted and reverse‐transcribed to cDNA using a Prime Script RT reagent kit (Vazyme) (Wang *et al*., 2018). The relative expression of a gene was determined by quantitative real‐time PCR in a CFX96 real‐time system (Biorad). The actin coding gene (accession number KP341672) was used as a reference and each experiment was repeated three times using a comparative *C*_t_ method as previously described (Sun *et al*., 2011).

### 4.10 Phylogenetic analysis

Multiple protein sequences were aligned by ClustalW program available in MEGA 5.0. The aligned sequences were performed using neighbour‐joining (NJ) analysis in MEGA 5.0 and the tree topology was evaluated with 1,000 bootstrap replicates (Li *et al*., 2016).

### 4.11 Yeast two‐hybrid and co‐immunoprecipitation

For Y2H assays, cDNA of each of the test genes was cloned into the yeast GAL4‐binding domain vector pGBKT7 and the GAL4‐activation domain vector pGADT7 (Clontech). The pGBKT7‐LAM and pGADT7 pair was used as a negative control and the pGBKT7‐53 and pGADT7 pair was used as a positive control. The pairs of Y2H plasmids were cotransformed into the yeast strain AH109. Transformants were grown on synthetic medium (SD) lacking Leu and Trp medium for 4 days, and then transferred to SD/−Ade−Leu−Trp−His medium and grown for 4 days at 30°C. At least three independent experiments were performed to confirm Y2H assay results (Liu *et al*., 2015).

A full‐length gene including its promoter sequence was amplified and cloned into the p1532‐GFP plasmid and pHZ‐FLAG plasmid and verified by DNA sequencing. The resultant plasmids were introduced into the protoplasts prepared from a Δ*Aatfb5* mutant. Transformants expressing fusion constructs were confirmed by western blotting with anti‐FLAG (Abcam) and anti‐GFP antibodies (Sigma). For Co‐IP assays, total proteins were extracted and incubated with the anti‐FLAG agarose (Sigma). Protein eluted from agarose was analysed by western blotting with anti‐GFP antibodies. The protein samples were also detected with monoclonal anti‐actin antibody (ABclonal Technology) as a control (Liu *et al*., 2015).

### 4.12 Statistical analysis

Data analyses and plotting were performed using SPSS statistics 19 (IBM) and Prism 5 (GraphPad). The significance of treatments was determined by analysis of variance and treatment means separated by Duncan's *t* test (*p* ≤ .05).

## Supporting information


**FIGURE S1** Identification and deletion of *Aatfb5*. (a) Phylogenetic tree of Tfb5 with the homologs from other species were constructed by the MEGA 5.0 program. (b) Alignments of amino acid sequences of Tfb5 proteins in *Alternaria alternata* and *Saccharomyces cerevisiae*. The characteristic stretches of hydrophobic residues are indicated by red boxes. (c) Schematic illustration of a double joint PCR strategy for disruption of the *Aatfb5* gene. (d) Image of DNA fragments amplified from genome DNA of Z7, two transformants and rescued strain with the primers indicated. Primers p7 and p8 were used to examine site‐specific integration of HPH within the *Aatfb5* allele. (e) Southern blot hybridization of genomic DNA from Z7, two putative disruptants and rescued strain

**FIGURE S2** Tfb5 is required for stress resistance. (a) Colonies of the wild‐type strain Z7, Δ*Aatfb5* and *Aatfb5‐c* on MM plates and MM plates containing 0.02% methyl methanesulphonate (MMS), 20 mM hydroxyurea (HU) and 0.1 mM cisplatin (CDDP). (b) Colonies of the Z7, Δ*Aatfb5* and *Aatfb5‐c* on MM plates and MM plates containing 10 mM hydrogen peroxide (H_2_O_2_), 0.05% diethyl maleate (DEM), 0.01% cumyl hydroperoxide (CHP), 2 mM VK3 or 0.05% *tert*‐butyl‐hydroxyperoxide (T‐BHP). (c) Colonies of the wild‐type strain Z7, Δ*Aatfb5* and *Aatfb5‐c* on MM plates and MM plates containing 100 µg/ml sodium dodecyl sulphate (SDS), 100 µg/ml Congo red (CR) and 200 µg/ml calcofluor white (CFW)

**FIGURE S3** HPLC analysis of ACT toxin purified from culture filtrates of Z7, Δ*Aatfb5*, Δ*Aacut2*, Δ*Aacut15* and Δ*Aacut2*Δ*Aacut15*. Arrows indicate ACT peaks

**FIGURE S4** Transformants on SD/−Leu/−Trp medium for 4 days at 30 °C

**FIGURE S5** Transcriptome analysis of differentially expressed genes (DEGs) in the *Alternaria alternata Aatfb5* deficiency mutant. (a) Volcano plot showing gene expression patterns in Δ*Aatfb5*. Red and green dots, respectively, represent the differentially up‐regulated and downregulated transcripts (*p* < .05, log_2_FoldChange > 0) in Δ*Aatfb5* compared to those of wild type. Blue dots represent the transcripts whose expression levels were not statistically different between two groups. (b) Circos plot displaying the differences in gene expression, and mRNA expression in Δ*Aatfb5* mutant compared to Z7. Each circle from the periphery to the core represents the following: chromosomal location, secondary metabolite gene clusters, differentially expressed genes (DEGs), down‐regulation in green, up‐regulation in red, and GC content. Gene duplications are shown in the centre. The conditionally dispensable chromosome (CDC) are including in this figure. (c) Gene Ontology (GO) enrichment analysis of DEGs between Δ*Aatfb5* and the wild type. Rich Factor represents the ratio of numbers of DEGs annotated in the GO term in relation to the numbers of all genes annotated in the same pathway. Only top 30 enriched GO terms are shown. (d) Scatter plot of KEGG pathway enrichment statistics based on DEGs in Δ*Aatfb5*. Rich Factor represents the ratio of numbers of DEGs annotated in the pathway term to the numbers of all genes annotated in the same pathway. Only top 20 enriched pathway terms are shown

**FIGURE S6** Identification and characteristics of Aacut2 and Aacut15 deficiency mutants. (a) Image of DNA fragments amplified from genome DNA of Z7, Δ*Aacut2*, Δ*Aacut15* and Δ*Aacut2ΔAacut15* strain with the primers indicated. Primers p17/p18 and p27/p28 were used to examine site‐specific integration of Neo/HPH within *Aacut2* and* Aacut15* allele. (b) Schematic illustration of a double joint PCR strategy for disruption of *Aacut2* and *Aacut15* genes. (c) Conidiation of *Alternaria alternata* strains

**TABLE S1** Oligonucleotide primers used in this study

**TABLE S2** Expression of SM genes in *Alternaria alternaria*

**TABLE S3** Expression of genes whose orthologs were associated with conidiation in *Alternaria alternaria, A. nidulans, Neurospora crassa* and* Magnaporthe oryzae*

## Data Availability

The raw sequence reads can be accessed at the NCBI SRA database at https://www.ncbi.nlm.nih.gov/sra with the accession numbers SRR11922316, SRR11922315, SRR11922314, SRR11922313, SRR11922312, and SRR11922311.
