# Supplementary figures and images for: The basal transcription factor II H subunit Tfb5 is required for stress response and pathogenicity in the tangerine pathotype of Alternaria alternata
Source: Mol Plant Pathol. 2020 Aug 10;21(10):1337–52. doi: 10.1111/mpp.12982 (PMC7488464; doi:10.1111/mpp.12982)

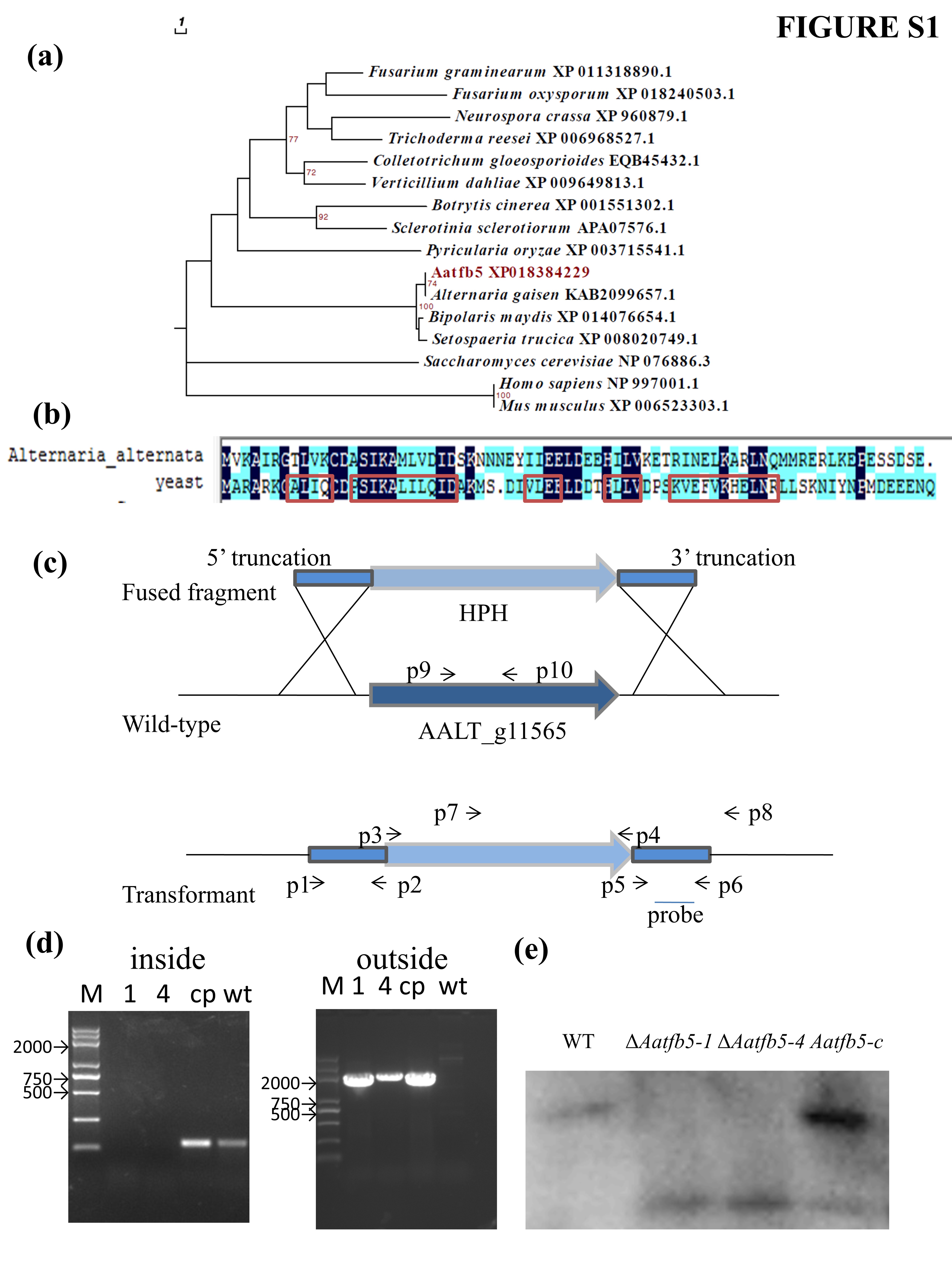

Supplement: Supplementary file 1 — FIGURE S1 Identification and deletion of Aatfb5. (a) Phylogenetic tree of Tfb5 with the homologs from other species were constructed by the MEGA 5.0 program. (b) Alignments of amino acid sequences of Tfb5 proteins in Alternaria alternata and Saccharomyces cerevisiae. The characteristic stretches of hydrophobic residues are indicated by red boxes. (c) Schematic illustration of a double joint PCR strategy for disruption of the Aatfb5 gene. (d) Image of DNA fragments amplified from genome DNA of Z7, two transformants and rescued strain with the primers indicated. Primers p7 and p8 were used to examine site‐specific integration of HPH within the Aatfb5 allele. (e) Southern blot hybridization of genomic DNA from Z7, two putative disruptants and rescued strain [file MPP-21-1337-s001.jpg]

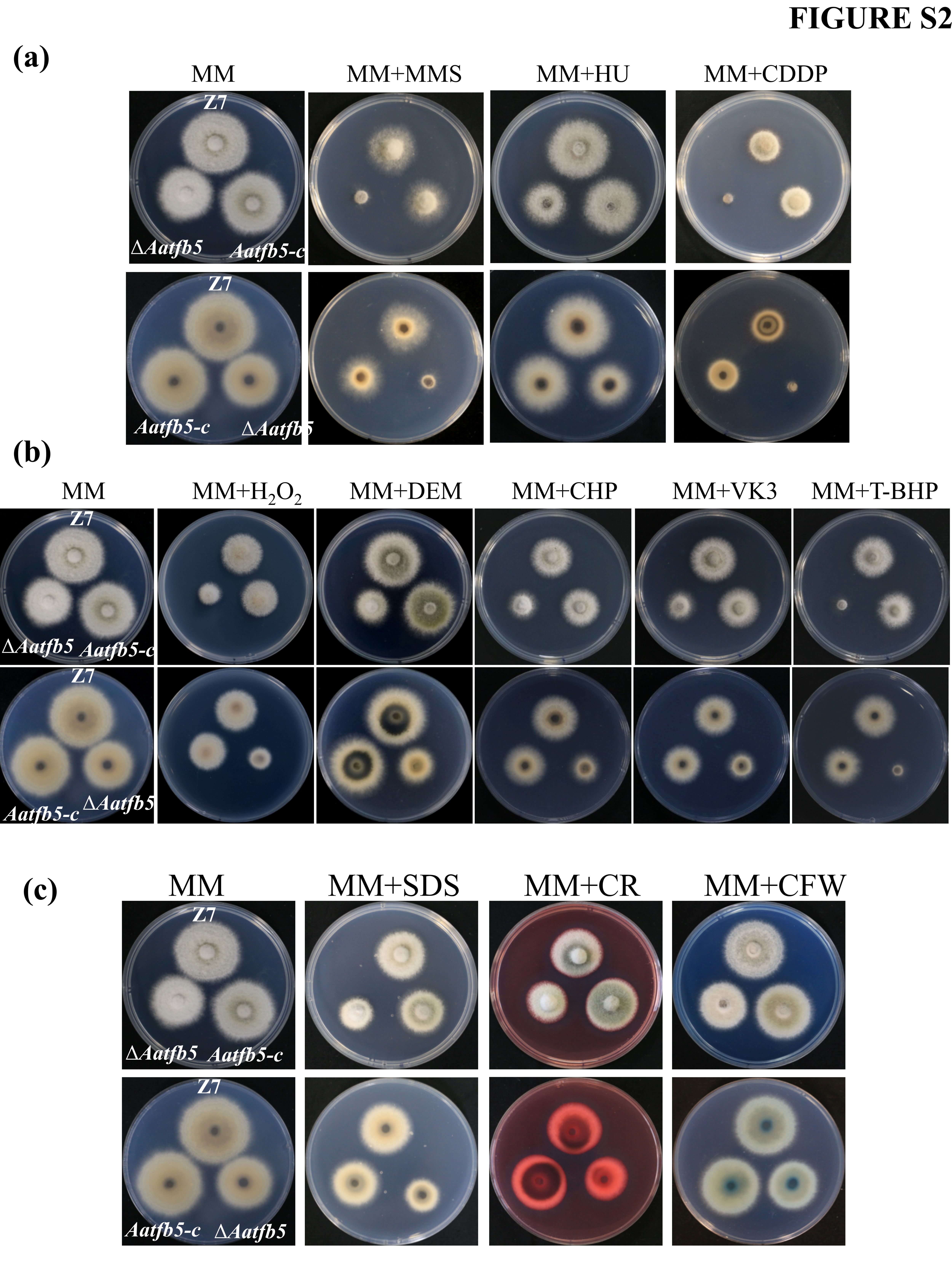

Supplement: Supplementary file 2 — FIGURE S2 Tfb5 is required for stress resistance. (a) Colonies of the wild‐type strain Z7, ΔAatfb5 and Aatfb5‐c on MM plates and MM plates containing 0.02% methyl methanesulphonate (MMS), 20 mM hydroxyurea (HU) and 0.1 mM cisplatin (CDDP). (b) Colonies of the Z7, ΔAatfb5 and Aatfb5‐c on MM plates and MM plates containing 10 mM hydrogen peroxide (H2O2), 0.05% diethyl maleate (DEM), 0.01% cumyl hydroperoxide (CHP), 2 mM VK3 or 0.05% tert‐butyl‐hydroxyperoxide (T‐BHP). (c) Colonies of the wild‐type strain Z7, ΔAatfb5 and Aatfb5‐c on MM plates and MM plates containing 100 µg/ml sodium dodecyl sulphate (SDS), 100 µg/ml Congo red (CR) and 200 µg/ml calcofluor white (CFW) [file MPP-21-1337-s002.jpg]

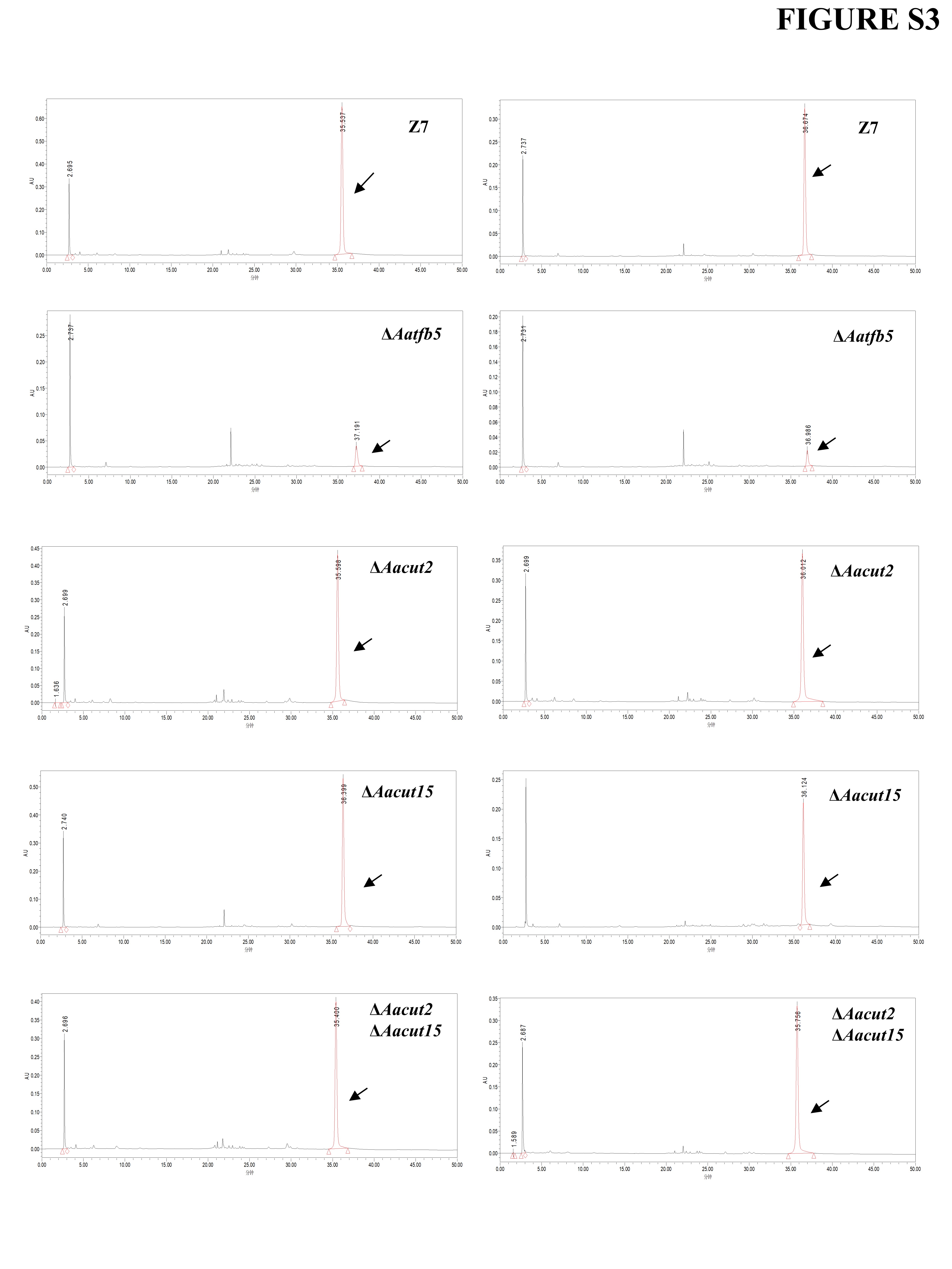

Supplement: Supplementary file 3 — FIGURE S3 HPLC analysis of ACT toxin purified from culture filtrates of Z7, ΔAatfb5, ΔAacut2, ΔAacut15 and ΔAacut2ΔAacut15. Arrows indicate ACT peaks [file MPP-21-1337-s003.jpg]

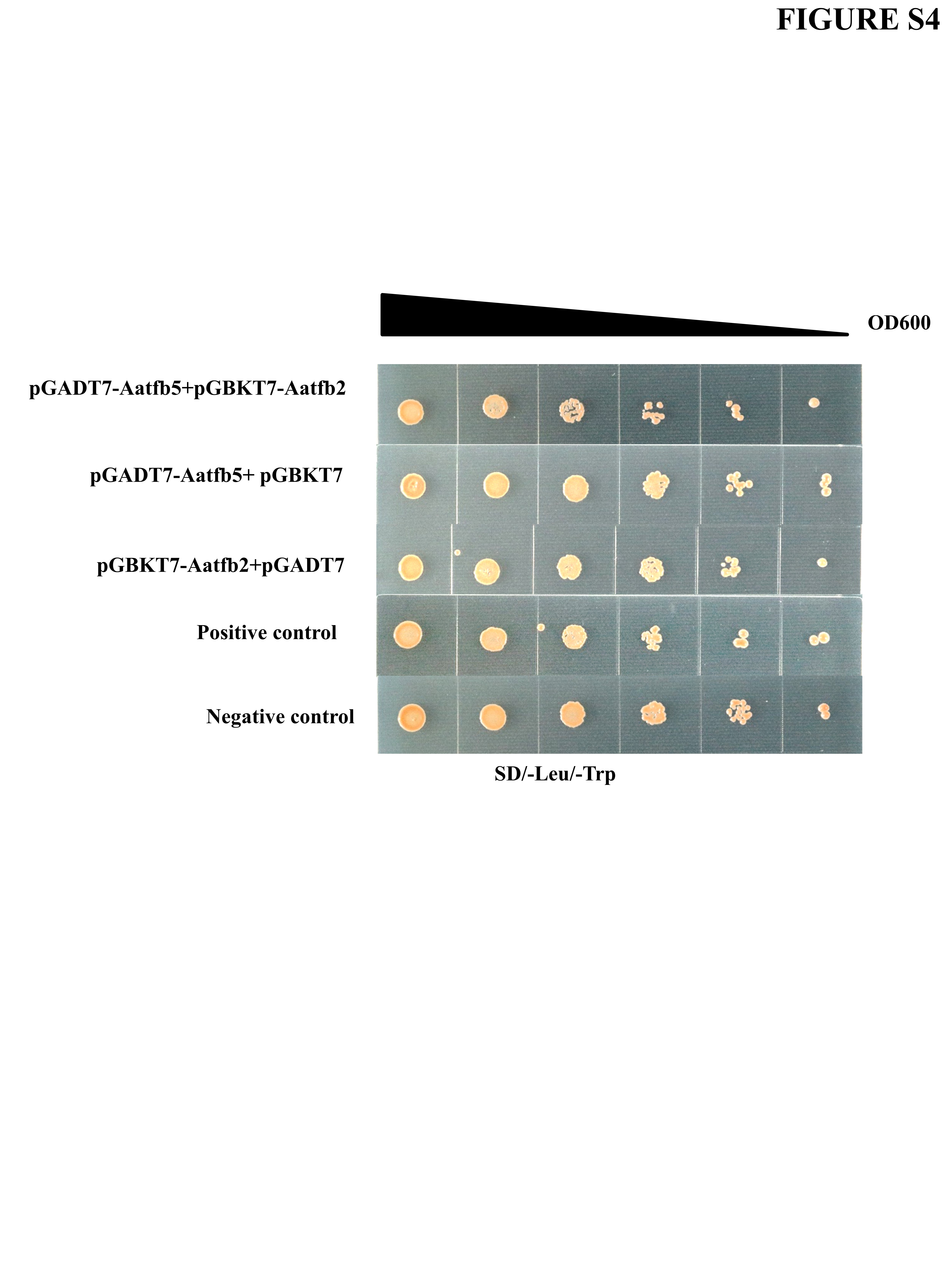

Supplement: Supplementary file 4 — FIGURE S4 Transformants on SD/−Leu/−Trp medium for 4 days at 30 °C [file MPP-21-1337-s004.jpg]

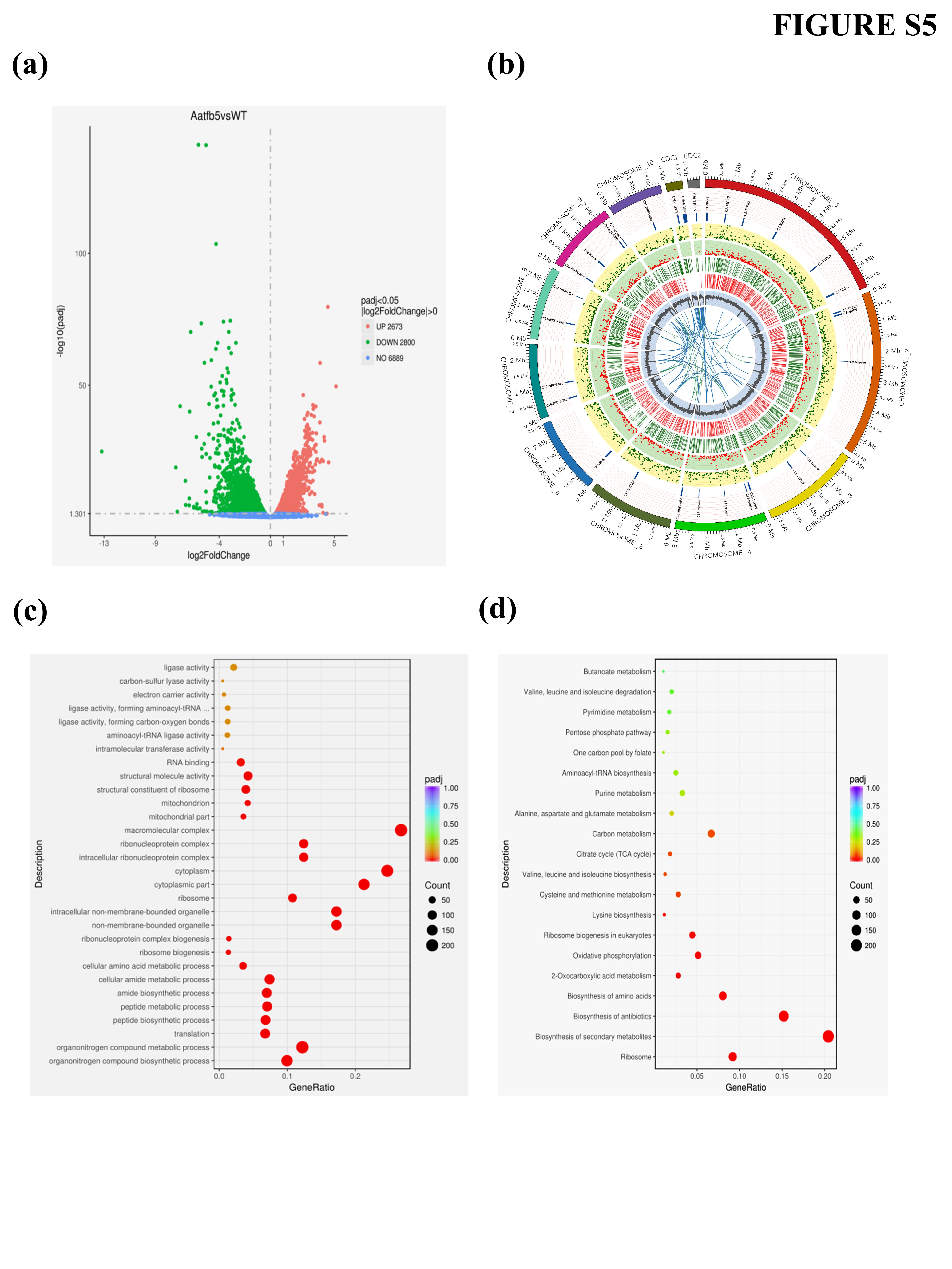

Supplement: Supplementary file 5 — FIGURE S5 Transcriptome analysis of differentially expressed genes (DEGs) in the Alternaria alternata Aatfb5 deficiency mutant. (a) Volcano plot showing gene expression patterns in ΔAatfb5. Red and green dots, respectively, represent the differentially up‐regulated and downregulated transcripts (p < .05, log2FoldChange > 0) in ΔAatfb5 compared to those of wild type. Blue dots represent the transcripts whose expression levels were not statistically different between two groups. (b) Circos plot displaying the differences in gene expression, and mRNA expression in ΔAatfb5 mutant compared to Z7. Each circle from the periphery to the core represents the following: chromosomal location, secondary metabolite gene clusters, differentially expressed genes (DEGs), down‐regulation in green, up‐regulation in red, and GC content. Gene duplications are shown in the centre. The conditionally dispensable chromosome (CDC) are including in this figure. (c) Gene Ontology (GO) enrichment analysis of DEGs between ΔAatfb5 and the wild type. Rich Factor represents the ratio of numbers of DEGs annotated in the GO term in relation to the numbers of all genes annotated in the same pathway. Only top 30 enriched GO terms are shown. (d) Scatter plot of KEGG pathway enrichment statistics based on DEGs in ΔAatfb5. Rich Factor represents the ratio of numbers of DEGs annotated in the pathway term to the numbers of all genes annotated in the same pathway. Only top 20 enriched pathway terms are shown [file MPP-21-1337-s005.jpg]

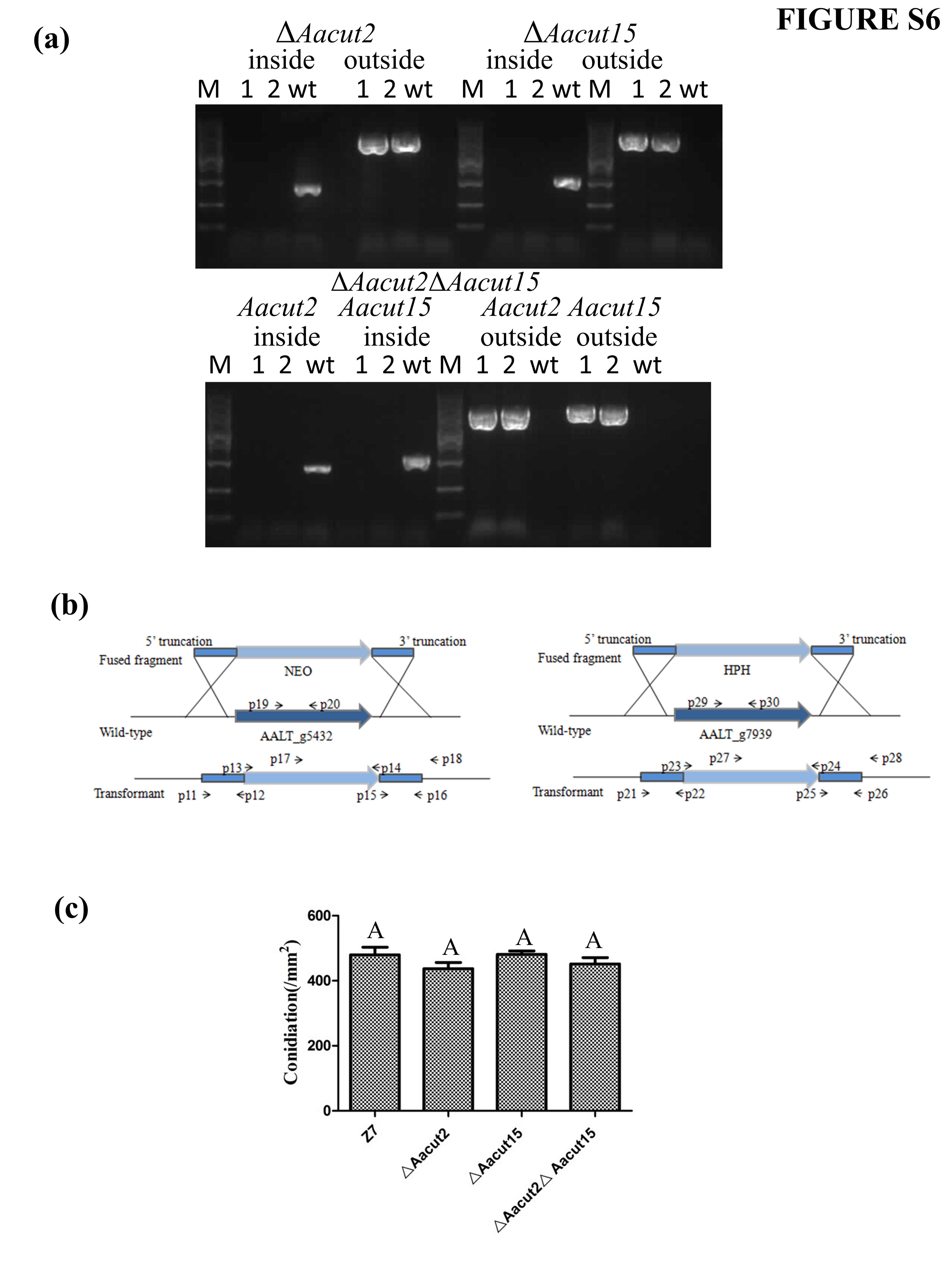

Supplement: Supplementary file 6 — FIGURE S6 Identification and characteristics of Aacut2 and Aacut15 deficiency mutants. (a) Image of DNA fragments amplified from genome DNA of Z7, ΔAacut2, ΔAacut15 and ΔAacut2ΔAacut15 strain with the primers indicated. Primers p17/p18 and p27/p28 were used to examine site‐specific integration of Neo/HPH within Aacut2 and Aacut15 allele. (b) Schematic illustration of a double joint PCR strategy for disruption of Aacut2 and Aacut15 genes. (c) Conidiation of Alternaria alternata strains [file MPP-21-1337-s006.jpg]
